# Alibis for Adult Play: A Goffmanian Account of Escaping Embarrassment in Adult Play

**DOI:** 10.1177/1555412017721086

**Published:** 2017-07-25

**Authors:** Sebastian Deterding

**Affiliations:** 1 University of York, York, United Kingdom

**Keywords:** accounts, adult play, adulthood, alibi, embarrassment, Erving Goffman, frame analysis, motivational accounting system, role distance

## Abstract

The social meanings of play sit at odds with norms of responsible and productive adult conduct. To be “caught” playing as an adult therefore risks embarrassment. Still, many designers want to create enjoyable, nonembarrassing play experiences for adults. To address this need, this article reads instances of spontaneous adult play through the lens of Erving Goffman’s theory of the interaction order to unpack conditions and strategies for nonembarrassing adult play. It identifies established frames, segregated audiences, scripts supporting smooth performance, managing audience awareness, role distancing, and, particularly, alibis for play: Adults routinely provide alternative, adult-appropriate motives to account for their play, such as child care, professional duties, creative expression, or health. Once legitimized, the norms and rules of play themselves then provide an alibi for behavior that would risk being embarrassing outside play.

“Play responsibly.”

–Slogan of many U.S. State Lotteries

## Introduction

A common saying has it that “play is the work of the child.” In contemporary Western societies, play is designated as a thing of children—and children alone. To our ears, “adult play” is either oxymoron or euphemism. Rarely do we see adults in the street engage in a bout of free play. And when we do, children or dogs seem necessarily involved. Why is that?

Across societies, in any given situation, adults are expected to competently and appropriately enact their social roles. Beyond direct sanctions, they are compelled to do so by embarrassment, a social emotion that internalizes the imagined devaluation of one’s self by others over incompetent or inappropriate acts (Goffman, 1967). Now even in societies less informed by a Protestant work ethic (Weber, 2010), play is considered the frivolous opposite of adult conduct (Sutton-Smith, 1997). Adults don’t play, it follows, because play would be role-inappropriate and therefore embarrassing. Indeed, there is ample evidence that feared embarrassment shies adults away from playing with toys (Heljakka, 2013), pervasive games and public installations (Wouters et al., 2016), even interactive museum exhibits (Scott, Hinton-Smith, Härmä, & Broome, 2013).

And yet, from Bingo halls to bowling nights and Adult Fans of Lego, adults *do* play, regularly so, and often quite unashamedly. This contradicts the strict juxtaposition of play and adulthood but also raises the question *when and how adults are able to engage in unembarrassed play*. This question holds direct value to designers who wish to reduce embarrassment as a hurdle to engagement or evoke embarrassment in more experimental, aesthetic works (Deterding et al., 2015). While the literatures on play (Henricks, 2015) and embarrassment (Miller, 1997) are rich and growing, there is as of yet little connection between the two, therefore little understanding of *how* embarrassment comes about in adult play—and thus, *how* designers might intentionally address it. To fill this gap, this article presents a theoretical account of the relation of adulthood, play, and embarrassment, grounded in Erving Goffman’s analyses of face-to-face interaction. His work is particularly apt for the task, as it provides a coherent theoretical framework of both play and embarrassment and is broadly congruent with current empirical data (Deterding, 2014; Miller, 1997). Like Goffman’s own accounts (Williams, 1988), the following analysis draws on everyday data, existing studies, and thought experiments to inform and constrain theory and illustrate ramifications. It first presents Goffman’s theory of the interaction order and embarrassment. It then unpacks how the social categories adulthood and play entail conflicting identity claims. From this, the article derives conditions and issues for nonembarrassing adult play as well as common techniques of deflecting embarrassment. The conclusion discusses emergent observations and open ends.

## Theoretical Framework

To survive, humans have to maintain the affection, benevolence, and trust of their conspecifics. The functioning of society in turn depends on people behaving intelligibly and reliably. The *interaction order* (Goffman, 1983) is the social machinery that ensures both in everyday life, and social emotions are its fuel (Goffman, 1967). The interaction order captures the social orderings that obtain in *response–present interaction*: when two or more people act in mutual reflexive perception and awareness (Goffman, 1983). Central to it is the “definition of the situation”: People make sense of actions based on what kind of situation they believe to be in. This sense-making relies on a shared repertoire of *frames*, reoccurring types of situations such as “going to the doctor” or “lecture” (Goffman, 1986). *Framing* is the usually taken-for-granted process by which actors constitute a situation as the instantiation of a specific frame. Frames differ from group to group and change over time. “Play” and “games” are everyday labels for a family of frames that share important meanings and norms (such as autotelic enjoyment) but also differ in many respects (Deterding, 2014).

Every frame entails a set of *roles* that can (and have to) be taken in by actors and defines what conduct is expected and appropriate for each role. During socialization, children internalize the frames and roles of their society—for example, how to be a proper “guest” when “going to a restaurant.” In the same process, they become aware of and construct their *self* from other’s responses to them across situations, become emotionally invested in this self, and adopt the values of their group regarding desirable self traits. In short, children start caring about *face*, “an image of self delineated in terms of approved social attributes” (Goffman, 1967, p. 5). *Social emotions* such as shame, pride, or embarrassment are the affective dimension of realizing or imagining how relevant others perceive and evaluate our self (Scheff, 2000; Turner & Stets, 2006).

Whenever individuals interact, they implicitly or explicitly project certain *identity claims* about their self: a competent cook, a kind nurse, or a polite passer-by (Goffman, 1967, pp. 105–108). These claims are mainly determined by the roles individuals occupy in a given situational frame (the groom at the wedding), but identity claims can obtain across situations: One doesn’t stop being (and having to be) a policeman, a daughter, or a gay rights activist at a wedding.

Whatever the specific roles and frames, the fundamental concern in any encounter is to maintain each other’s face, chiefly by projecting and regarding valued and appropriate identities, fulfilling expectations connected to them, and keeping interaction flowing smoothly. As a social event, *embarrassment* occurs when the valued identity claims participants project in an encounter are somehow disconfirmed (Goffman, 1967, pp. 97–112). Such disconfirming also disrupts the smooth flow of interaction: It creates a pause where everyone is torn into self-conscious awareness of the disruption with no ready script how to “move swiftly on,” restore lost face and get interaction going again. This in turn disconfirms every participant’s basic valued identity as a benign and skilled interactant: Adults *should* “know not to embarrass each other” and “know what to say.” As an internal process, embarrassment is the sudden self-conscious awareness of such identity-disconfirming events, together with negative affect and arousal over seeing or imagining others who disapprove of us as a result. This motivates us to avoid embarrassment-prone situations and appease after an inappropriate action: blushing, apologies, and making amends all signal that the embarrassment was unintentional, that we feel bad about it, and can therefore be trusted to act competently and considerate in the future (Goffman, 1967, pp. 113–114). All this makes embarrassment an important mechanism of social control.

In sum, embarrassment is overtly an event that publicly discredits active valued identity claims of participants in an encounter. It discredits identities of the embarrassed (e.g., being exposed in a white lie), the embarrasser (being exposed to be so inconsiderate as to let the truth slip), and possibly, all participants (being exposed as unskillful interactants not knowing how to get the conversation going again). Covertly, embarrassment is self-conscious awareness of and negative affect and arousal over the resultant (feared, imagined, and observed) devaluation of one’s self by present or imagined others.

## The Identity Claims of Adulthood and Play

Goffman’s theory not only initiated sociological and psychological research on embarrassment: It is still in active use and congruent with current empirical data (Miller, 1997; Turner & Stets, 2006). Applying it to adult play immediately raises the question what identity claims are entailed in adulthood and play.

Like many cultural dualisms, adulthood is socially defined in contradistinction to childhood (Jenkins, 2008, p. 257). Beyond biological maturity, it marks the social status of being a “full” member of society as a “rational, objective, productive individual” (Moran, 1979, p. 24). According to contemporary (U.S. American) folk conceptions, becoming adult means becoming *responsible* (Arnett, 1998, 2001). Adults are expected to fully and independently assess the ramifications of their actions against their own stable values and commitments, and self-regulate their conduct based on that. Second, adults are expected to be *independently dependable*. They should develop the capacity to sustain themselves and a household of (inter)dependent partners and children. Third, adults are expected to *self-determinedly comply with social norms*, without sanction or reminder. In short, the identity claim “adult” entails showing stable internal values and commitments; capable self-governance based on those mindful of others and social norms; and a capacity and concern for sustaining oneself and one’s dependents (Arnett, 1998, 2001).

How does this sit with the social meanings of play (Heljakka, 2013, pp. 200–235; Sutton-Smith, 1997)? Where adulthood stands for stable values, personality, and conduct, play signifies the Protean exploration of possible goals, selves, and behaviors. Play submits adult self-governance to arbitrary outer rules and the spontaneous flow of events. Adulthood puts responsibility for collective survival first (via productive work and social care), while play follows the selfish pleasure principle, an “occasion of pure waste” (Caillois, 2001, p. 55) that temporarily abdicates concerns for instrumental outcomes. Where adulthood means reliable, norm-abiding conduct, play signifies the immoderate, spontaneous, norm-breaching, always on the brink of dark play (Linderoth & Mortensen, 2015). Finally, the main socially acknowledged function of play is to prepare for adulthood (Sutton-Smith, 1997). Hence to *be* adult is to *not be* in need of play anymore. All this is not to deny that there are abundant empirical instances of productive, regulated, and norm-abiding play. What matters here are the *meanings* ascribed to play in contemporary Western societies and, thus, the *identity* projected by overt acts of play.

## Conditions of Legitimate Adult Play

Adulthood and play, then, claim fundamentally divergent identities. When we say that play is childish, we mean that it is irresponsibly pleasure-driven, unproductive, and not mindful of social norms. Given this clash, how do we explain unembarrassed adult play in the wild? If identity-*incongruent* acts embarrass, play should not embarrass adults when it is identity-*congruent*: When to play *is* to be an adult, predictable, responsible, productive, norm-minding member of society. For instance, it is the kindergarten teacher’s adult job to play Patty Cake with children for their edification; to *not* do so while on duty would be inappropriate, as would be playing Patty Cake with adult strangers on a subway for fun. More generally, adult play should not embarrass when it presents the smooth, unselfconscious performance of appropriate identities as part of established social frames and roles. Let us unpack these conditions.

### Established Frames

First, we require an institutionalized frame within a particular social group: For instance, a shared understanding of “baseball” being “a thing,” and a thing adults can legitimately engage in. Else, one’s behavior would be unintelligibly (and embarrassingly) strange. Beyond basic frames, people can *reframe* or “key” a given strip of activity, for example, do something ironically (Goffman, 1986, pp. 43–74). While play itself may not be appropriate, its keying can be. Gambling is stigmatized, yet an actor can play a gambler in a theater production unspoilt—if she manages to signal sufficient difference between role and person.

Frames legitimate play for *particular persons* in *particular roles* in *particular situations*. Playing on your mobile phone to pass time is fine for a commuter on a subway but not for a member of parliament during debate (Cresci, 2016). Continuing biographical identity claims may clash or align with play across multiple situations. For many, cross-dressing for a role in a school play embarrassingly disconfirms their valued gender identity.

One broad family of legitimate adult play frames are *professional and social responsibilities*. Parents, teachers, caretakers, therapists, trainers, game journalists all play in direct service of the well-being and education of others. For them, to play is to provide. Athletes and actors compete and perform for public edification and individual gain. Game and toy designers and scholars play to test, improve, and understand play as a professional subject matter. Such legitimate *instrumental keyings* of play-as-work (Deterding, 2014, pp. 325–377) have also been called “play-to-order” (Stenros, 2015b, p. 93).

A second family is *leisurely recreation*. The adult demands of constant self-regulation, role conformity, and self-provision are socially recognized to be taxing. To cope, restore, and realize themselves as human beings, adults are allowed “activity enclaves” (Cohen & Taylor, 1976, p. 97) where they can temporarily cop out of the other-determined routines of everyday life, lower self-regulation, engage in hedonic pursuits, and express and explore alternate identities and practices not fitting their official roles. Common instances are hobbies, games, mass media consumption, travel, or festivals (Cohen & Taylor, 1976, pp. 77–137; Ravenscroft & Gilchrist, 2009). Mardi Gras is a well-studied example where out-of-character sexual behavior is framed as playful and accepted in public daylight (Redmon, 2003).

Notably, such enclaves aren’t any *less* socially regulated, only *differently*. Each of these “institutionalized escapes…*has its time and its place*” (Cohen & Taylor, 1976, p. 113): They are frames with particular normal (and normative) motives, settings, objects, roles, scripts, and boundaries. Our escapes from the bounds of routine and convention are themselves highly bounded, routine, and conventional (Cohen & Taylor, 1976, p. 97). Like all frames, they come with limits that can be permissibly framed by them (Goffman, 1986, pp. 49–52, 56). Many consider representing the holocaust in a game as inappropriately trivializing (Chapman & Linderoth, 2015). Importantly, adult leisurely play is expected to *subordinate* to work and social responsibilities. Leisurely gaming takes place during evenings, weekends, or holidays, when all other obligations are “done for the day” or in “empty times” like a subway commute (Deterding, 2014). Two contributions in the present special issue directly testify to this subordination norm: Group sex events are scheduled in advance to allow participating parents to arrange babysitters (Harviainen & Frank, 2016, p. 10); board gaming parents fit their hobby into their parental duties by playing with their children or shifting gameplay to times when the children are in bed (Rogerson & Gibbs, 2016). Being caught breaking these norms may induce embarrassment or shame. Failing to even acknowledge their existence harbors a worse fate—being considered insane (Goffman, 1983a), as the following statement by one of Rogerson and Gibbs (2016, p. 12) interviewees illustrates: she “felt that her ex-husband’s insistence on continuing to host and participate in game nights despite having a young baby was a factor in their marriage breakdown. She saw this insistence as a symptom of her former partner’s mental health problems—‘he just wouldn’t grow up’.”

Different groups share different play frames and valuations of them. What “official” society considers inappropriate can be appreciated in subcultures and vice versa (Copes & Williams, 2007). Emotional distress that “bleeds” into postgame reality directly violates the norms of contemporary leisurely gaming frames (Deterding, 2014). And yet, in the Nordic Larp community (Stenros & Montola, 2010, pp. 20–28), which considers live action role-play (larp) a valid artistic medium for insightful and transformative experiences, larps like *Gang Rape* are positively valued for their ability to cause such “bleeding” distress (Montola, 2010).

Finally, different forms or genres of play are differently valued for different persons. Sports and family board gaming are broadly acknowledged as “valuable” pastime, while gambling carries stigma (King, 1990). Toy play is highly inappropriate for adults, hence gets hidden or reframed as “collecting” (Heljakka, 2013). Outside role-playing game communities, adult pretend play has to stay in the imagination, cued by fictional media or carnival props but not publicly acted out in extenso (Cohen & Taylor, 1976, p. 77). Even (or particularly) subcultural groups that hold adult play in high regard—such as fantasy/science fiction fandom and geekdom—maintain finely tuned and gendered status hierarchies of play genres, with writing commercial fiction at the top and Furry cosplay at the bottom (Busse, 2013).

### Effective Framing

An established frame is necessary but not sufficient for nonembarrassing adult play: *framing* a strip of events as an instantiation of such a frame requires necessary though typically taken-for-granted work and resources. “Baseball” needs to be practically arranged (bats, gloves, balls, knowledgeable players, the works), but also signaled. Embarrassment ensues where people engage in misframings (Goffman, 1986, p. 302) such as mistaking a serious remark for a joke, or where framing remains ambiguous, for in such cases, observer and observed may feel to appear alternatively thick for “not getting it” or strange for doing something inscrutable.

The more institutionalized and conventionalized a play frame, and the more conventional the materials and actions of the current strip, the more readily their framing. Novel play forms and unconventional instantiations therefore run higher risks of being perceived as improper or strange. Explicit framing indicators or “brackets” can reduce such risk (Goffman, 1986, p. 254). For instance, pervasive games (Montola, Stenros, & Waern, 2009) occur in public space commonly *not* used for play and are unknown to the broader population as a genre. “Festival” in contrast is a well-instituted frame for temporary extraordinary behavior in public (Ravenscroft & Gilchrist, 2009). Hence, pervasive games are commonly arranged as part of explicit festivals such as *Playpublik*^1^ that employ brackets like police tape and signposts stating that a festival is in progress to help passers-by make sense of the unfolding events.

### Smooth Performance

By taking on the role of a player, people effectively claim to be reasonably apt ones. Public performance frames such as sportive or creative contests raise the stakes of such skilled performance, as they explicitly invite onlookers to evaluate performers by it. Stepping up for a round of Karaoke effectively claims not just basic but *performance-worthy* singing skills, which may be one reason many shy away from it. Taking on a player role also claims basic know-how for the play form in question—another reason why novel play forms are embarrassment-prone. Imagine a subway conductor suddenly invited to a pervasive game in a subway. A second ago, she had expert knowledge regarding frame (subway ride) and role (conductor). Imposing play on her puts herself at risk: torn out of routine and with no knowledge of the game, exposed to a public that might find her breaking professional role by joining in. All she is left with is choosing between the embarrassment of being a stiff spoilsport and the embarrassment of being a clumsy novice and neglectful professional.

This also explains why improvisational or “free,” paidic play (Caillois, 2001) triggers particular embarrassment fears. Games facilitate smooth interaction. At every turn, a player can fall back on predefined, ready-to-hand goals, rules, and actions that are immediately intelligible to all thanks to their shared game knowledge. Improvisation offers no such scripts to follow or hide behind, making embarrassing gaffes and pauses more likely (Lee, 2009), and every act can be read as direct expressing one’s personality, desires, and creative capacity. Professional improvisers like rappers therefore train “canned resources” (Lee, 2009) or prepared scripts to save face and maintain the flow of interaction during inspirational blanks.

### Unselfconscious Involvement

Every frame and role hold norms how deeply to be visibly involved in a particular focus (Goffman, 1967, pp. 114–117). Adults—particularly males and members of high-risk professions—are expected to maintain a certain “cool” poise and distance across situations (Goffman, 1967, pp. 214–238). Embarrassment becomes likely when play becomes so under- or overinvolving that participants can’t hold back inappropriate involvement expressions (falling asleep and shouting in triumph); when outsiders peek in on the proceedings, thus actualizing adult role norms of cool composure; or when uninvolving activity releases awareness into self-conscious thought about one’s public appearance. The flustering and blushing produced by this may then cause further embarrassment by disrupting one’s smooth and comported conduct (Goffman, 1967, pp. 101–102). Thankfully, play frames present the rare case where deep unselfconscious involvement is both socially expected *and* afforded by play objects and practices (Deterding, 2015b).

## Strategies of Embarrassment Deflection

Even legitimate adult play may provoke embarrassment, be it that structural conditions make it inevitable (Goffman, 1967, pp. 110–113), be it that the tension between participant identities and the particular play frame and genre is too strong (Goffman, 1986, p. 274). Straightforward avoidance is a common response (Goffman, 1967, p. 15). Yet sometimes, individuals cannot or don’t want to evade play. Instead, they will look for strategies to reduce embarrassment (risk), something which professional play practitioners should be especially aware of and adept at (Lee, 2009). With no claim to comprehensiveness, four sets of strategies are worth highlighting: alibis, audience, awareness management, and role distancing.

### Alibis

Whether an action disconfirms an identity depends on what meaning people ascribe to it: Was that elbow buff intentional or accidental? Hence, the *accounts* people offer for actions are often as crucial as the actions themselves (Orbuch, 1997). A fundamental norm of adult human conduct is to be reasonable. To not appear insane, our behavior has to be intelligible as guided by reasons, ideally “good” ones (Goffman, 1983a). Whenever others find our behavior questionable, they will likely hold us *accountable*: Asking us to provide an account why we did what we did. To maintain social standing and smooth interaction, we often have reason to give reasons for our actions different from the actual ones (Mills, 1940). In doing so, we can rely on (and ought to limit ourselves to) “motivational accounting systems” (Ben-Yehuda, 1990), the set of reasons accepted as normal for a particular activity.

Adult play presents the interesting case where accounting not just has to render play reasonable *somehow* but also in a way that neutralizes its tension with adult identity. Autotelic enjoyment is widely recognized as the prime motivation of leisurely play (Boyle, Connolly, Hainey, & Boyle, 2012). And yet, “pure” autotelic enjoyment sits at odds with adult identity claims of responsibility, productivity, and self-regulation. When held accountable for their play, adults are therefore likely to find themselves in need of an *alibi*.

In legal and everyday language, an alibi is “a plea by a person accused of an act that he or she was elsewhere when it took place”; in a weaker and more colloquial sense, it means “an excuse, pretext, or justification” (OED Online, 2015; Olson & Wells, 2004). In game scholarship, “alibi” has been used to denote how the rules of a game and social contract of gaming give a pretext to engage in behaviors that would be considered inappropriate out of game, such as touching people during a game of Twister (Montola, 2010; Poremba, 2007; Stenros, 2015a). Grounded in the present analysis, one can theorize alibi more broadly as *a motivational account that deflects negative inference from displayed behavior to a person’s identity*. The account can be factually true or not, explicit or implicit: What matters is that in the course of events, it provides effectively implied plausible deniability. The fact that constructing a model train with your child is “quality family time” and “a great gift” deflects the inference that one might secretly still want to play with model trains.

Irrespective of actual motivations, when adults are faced with an audience that may find play at odds with their adult identity, they will be prone to account for their play with such alibis. Gaming consoles are “really” bought because of the in-built DVD player (Thornham, 2009). Wargames train valuable cognitive faculties (Dunnigan, 2000). Board gaming facilitates family cohesion (Rogerson & Gibbs, 2016). Role-playing nurtures creativity, self-development, and social skills (Bowman, 2010). Toys are smart investments in “collections” that accrue exchange value (Heljakka, 2013). Playing is good for your health (Brown, 2009). Adult coloring books are “Anti-Stress Art Therapy for Busy People” (Farrarons, 2015); and so on. Lavenir’s (2015) ethnography of a French video-gaming workshop for female seniors provides an instructive example. Among themselves, these women admit and indeed insist that video gaming is an autotelic pleasure, not a means to a productive end like cognitive training—also to deflect the stigma that they would be so old as to need training. Yet outside the workshop, they either hide that they play video games at all, or “when confronted with someone coming from outside […], fall back on a carefully crafted discourse on their practice and motivations” (Lavenir, 2015, n.p.).

Amateur gamblers peruse opposite alibis for the same reasons: They account for gambling *as* inconsequential leisure because the gambling stigma of addiction and financial folly far outstrips the mild disapproval stirred by “frivolous fun” (Smith & Preston, 1984). And like professional gamers, professional gamblers neutralize the stigma of gambling by accounting for it as routine, professional, and gainful labor (Vines & Linders, 2016).

The same alibi logic operates in the ubiquitous discourses of game designers and scholars that frame games in terms of their economic import; educational and health benefits; value as an artistic medium; or potential to drive collaboration, creativity, and productivity. On the one hand, their profession provides them ready alibis for play. As the half-joke among game scholars goes: “This is all research.” On the other, having their professional selves invested in a “frivolous” pastime still exposes them to potential disapproval in many circles. In stressing the “serious” positive social, economic, and cultural effects of play, game scholars and designers not only institute new alibis. They effectively operate as moral entrepreneurs who normalize and even valorize play—and with it, their own professional identities (Best, 2003, pp. 38–43). Yet in doing so, they implicitly reinforce the norm that “just having fun” is not reason *enough* for adult play, that play and enjoyment *ought* to be subservient to productive and social rationales.

**Figure 1.**
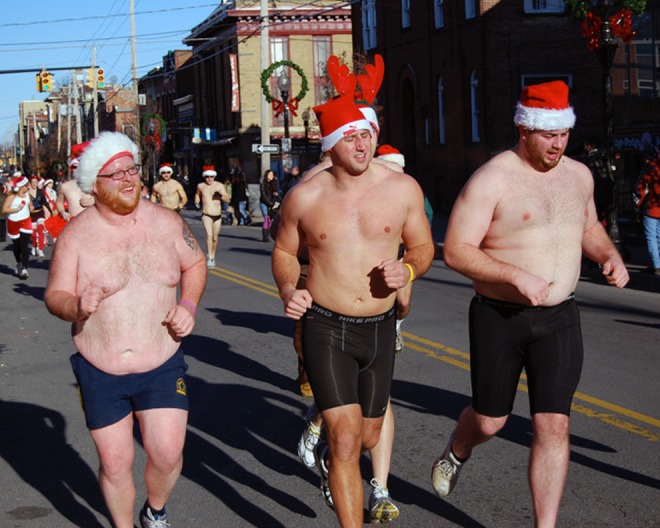
Alibis for play: At the annual Santa Speedo Run, adults run in nothing but speedos and Santa hats through Boston’s winter to raise money for children. The overt “good cause” reframes an otherwise embarrassingly strange, even criminal offence into ennobling care for others. “Santa Speedo Sprint 2008: The Bear Club for Men” © by Tim Schapker, https://www.flickr.com/photos/albany_tim/3113422672.

A second source of embarrassment-deflecting alibis are keyings. One example are overt *regroundings* (Goffman, 1986, p. 74), “the performance of an activity [….] for reasons or motives felt to be radically different from those that govern ordinary actors”. Running through winter streets in speedos is strange; doing so *for charity* as part of the “Santa Speedo Run”^2^ is not (Figure 1). The more excruciating the embarrassment endured for a “noble” cause, the more positively it reflects on the person. Another alibi-providing keying are humorous or ironic reversals. An embarrassment is an *un*intentional breach of normative expectations that is therefore read as a direct, *non*fabricated signal of a person’s lacking skill, character, or goodwill. Conversely, humor is a benign *intentional* breach of normative expectations (McGraw & Warren, 2010) that is therefore read as *fabricated* and thus not revealing of a person’s actual skill, character, or goodwill. If anything, it reveals the benign intent of humoring others. Humor entails a risky identity claim itself, of course—few things are quite as embarrassing as a failing joke—but it is nevertheless a common saving strategy for embarrassment. By following up a faux-pas with a witty remark that doubles down on it, we invite observers to retrospectively frame the mishap as part of an intentional attempt at humoring: “It is natural, then, to find embarrassment and joking together, for both help in denying the same reality” (Goffman, 1967, p. 112).

Such humorous meaning reversals can also be employed preemptively. If tasked with an action that is likely to discredit identity claims, people often make a point of mock performing, artificially exaggerating, or otherwise ironically keying the action. Afraid of embarrassing themselves at Karaoke, people might choose an intentionally campy song and sing ostentatiously false and with ostentatiously dramatic gestures to clarify that whatever expression they might produce, these are *intended* to be entertainingly bad, not due to lacking vocal or bodily grace.

**Figure 2.**
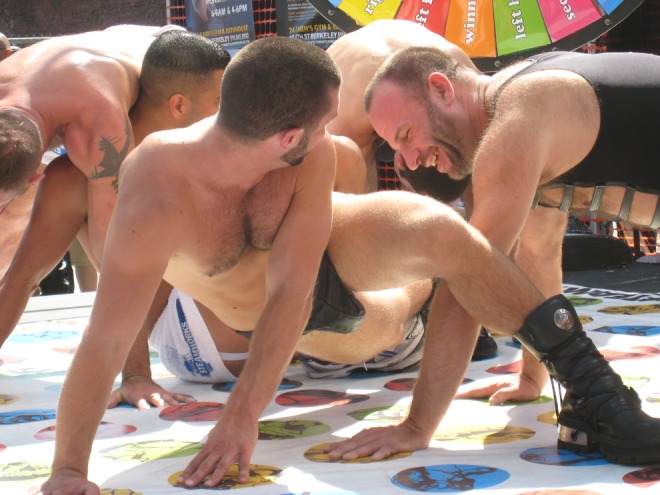
Alibis of play: Fulfilling the role expectations of being a considerate player requires people to play by the rules. The norms of Twister thus temporarily replace the public interaction norm not to touch strangers. In addition, touching another romantically always carries the risk of embarrassing rejection. Twister gives an alternative account to deflect this risk: “the rules made me do it.” “I want to play twister” © Jay Petersen, https://www.flickr.com/photos/fuzzyjay/2899687311, licensed under CC BY-NC-SA 2.0.

In fact, once we successfully established an alibi *for* play (“games are educational”), the play framing itself provides an alibi *of* play *for* in-play activities. The mad-lib rules and offensive cards of *Cards Against Humanity* “provide[.] permission to tell jokes you don’t dare by removing all sense of responsibility” (Shut Up & Sit Down, 2015). On a positive note, people often actively seek out and play with the risk of embarrassing each other, and typically so in a play form like teasing: Few things are quite as involving as other human beings putting their selves on the line (Goffman, 1953, pp. 319–327). Jointly embarrassing ourselves nonseriously builds trust and levels status differences. The nonserious frame of play provides an alibi for making advances; showing off desirable traits like humility, composure, cool, and social grace (Goffman, 1967, pp. 214–239); or voicing serious demands and grievances while saving face for everyone (Keltner, Capps, Kring, Young, & Heerey, 2001). To desire or brag or reprimand openly would fully expose everyone to potential embarrassment; to do so playfully offers the plausible deniability that all was meant in jest. In practice, alibis for and of play often readily fall together. *We* didn’t *choose* to play Twister, the host suggested it as an icebreaker, and *we* totally *don’t* enjoy being entangled right now, the rules demanded it: We’re having fun while saving face (Figure 2).

### Audience Management

In the course of our lives, we have to claim multiple identities and satisfy multiple role expectations with often conflicting demands. Likewise, we traverse groups with different norms. What is normal to our geek friends is strange to our families at home. Most everyday encounters luckily only demand the performance of *select* identities before *congruent* audiences: a commanding dom at home, a nurturing teacher at kindergarten (Goffman, 1967, p. 108). However, certain situations “collapse contexts” (boyd, 2008) and with them, identities and audiences, making embarrassment unavoidable (Goffman, 1967, pp. 109–113). The birthday party, Facebook feed, or intercultural business meeting are ready examples. This makes *public* play structurally embarrassment-prone. Public spaces expose us to diverse and usually not fully overseeable audiences, carrying the (perceived) danger of doing *some*thing that breaks the norms of *some* incidental observer. *Audience segregation* is therefore a common strategy of deflecting embarrassment (Goffman, 1967, p. 108). It is an elusive obvious fact that adult play typically takes place in private home spaces where one is literally shielded from the eyes and ears of potentially disapproving others (Deterding, 2015b). Embarrassing play activities like Karaoke are staged in dedicated places not transparent to the outside world, and groups rent individual private cabins within them.

Just like nonembarrassing play needs the absence of disapproving others, so it is facilitated by present approving ones. The framing of a strip of events as legitimate play requires response-present others to acknowledge its reality through joint enactment or accepting inattention (“nothing special to see here”). To consider an activity “normal,” a sufficient number of others has to explicitly or implicitly signal that it is normal (Goffman, 1972, p. 72). As threshold models of collective behavior like rioting predict, actors will engage in a behavior once the proportion of perceived others engaging in it tips the benefits minus costs of engaging over those of not engaging (Granovetter, 1978; Oliver, 1993). Given enough dancers on the dance floor, the risk of being seen wrongly claiming performance-worthy dancing skills becomes smaller than the risk of being spotted as a spoilsport on the edge (leaving potential enjoyment benefits of dancing aside for the moment). As individuals may have different thresholds, cascade dynamics become likely. The moment one very low-threshold dancer “breaks the ice,” their social proof may be enough to tip people with the next higher thresholds over the edge and so on. This dynamic is recognized as one aspect of the so-called honeypot effect (Wouters et al., 2016). Other people visibly engaging with a public interface stimulate passers-by to likewise approach, observe, and engage with it. Put differently, *audience aggregation* is another relevant strategy for deflecting embarrassment. Festivals for instance aggregate a self-selecting group of people into spatiotemporal response-presence who feature highly approval of and low engagement thresholds for the celebrated play activity. Surrounded by hundreds of other cosplayers in costume, we feel normal in our faux-troll second skin.

### Awareness Management

Physically removing audiences is one way of deflecting their perceived disapproval. Another is to interrupt the mutual perception between actor and audience: Be it that the audience is absent in the actor’s awareness, be it that the actor is visibly absent in the audience’s. The latter is another embarrassment-reducing dynamic of large group play. The more people there are in a street game, the less the individual has to fear that disapproving eyes fall on her in particular. Dark rooms, costumes, masks, pseudonyms, and avatars also serve this function. They make actors visible but not *identifiable*. During the immersive theater productions of Punchdrunk, I observed many uninhibited behaviors by mask-wearing audience members. Similarly, concerns for embarrassment recede when individuals become engrossed in an activity. In personal observation, when adults try out VR gaming for the first time, they first show high anxious awareness of how silly they must look wearing a headset and making strange gestures, but as goggles and headphones cut off perception of the audience and gameplay demands their full attention, this anxiety vanishes, only to return with shocked realization when they take headset and headphone of and perceive their (often laughing) audience.

### Role Distancing

A final deflection strategy is *role distancing*, “actions which effectively convey some disdainful detachment of the performer from a role he is performing” (Goffman, 1972, p. 110). Peterson (2011, pp. 145–170) documents a nice instance. When she asked two young adolescent boys to play the “girl game” *The Sims 2*, they immediately managed the identity threat by keying into nonserious, parodist play, creating the to-them-absurd characters of an overweight, Black homosexual couple. With smiles, giggles, expletives, gesturing, and mimicking, they constantly signaled how ridiculous and mock-offensive all this play is.

## Conclusions and Elisions

Embarrassment arises when people become aware of imagined or real others perceiving events disconfirming their valued identities. The social meanings of play—unruly, pleasure-driven, free, and unproductive—disconfirm the valued social identity of being a self-regulated, norm-abiding, and productive adult. Hence, play risks embarrassing adults. And yet, there are instances when adults *do* play unembarrassedly. To resolve this paradox, we unpacked conditions and strategies for unembarrassed adult play. Unembarrassed play requires an established frame of legitimate play; we found two families of such frames, professional responsibilities like play-as-child care and leisurely recreation like hobbies. It requires that valued identities and situational role expectations don’t clash, which is facilitated by physically segregating audiences with differing expectations or by enacting distance to a clashing situationally demanded role. It requires clearly framing the given activity as an instance of a legitimate frame, supported by physical framing signals and aggregating audiences that mutually signal the normalcy of play by joining in, cheering, or not minding. It requires smooth performance of role expectations, made easier by clear scripts, rules, and fallback routines. It requires appropriately deep involvement in play and lowered self-consciousness of one’s public appearance, aided by engrossing activities and masks, pseudonymous avatars, or hiding in a crowd, disrupting the response-presence of others.

Finally, we found that the most obvious motivation for play—autotelic enjoyment—also sits in most direct tension with adult identity. To account for their play, adults therefore regularly resort to *alibis*, motivational accounts that deflect negative inference from their play behavior to their character. Adults account for play as *serving* their adult responsibilities: be it that it is part of gainful labor or providing for others; be it that it nurtures their health and productive faculties; be it that it serves communal cohesion or artistic expression; or be it that it is re-grounded as charity or a joke. To play as an adult—legitimately, unembarrassed—is to play responsibly. As one of the countless guidelines for “responsible gambling” puts it: “Don’t let gambling interfere with family, friends or work.”^3^

This observation underlines the general methodological caveat that why people *do* things and why they *say* they do things are often different (Briggs, 2007; Mills, 1940)—a caveat rarely heeded in contemporary research on gameplay motivation that takes participants’ statements at face value (Boyle et al., 2012, pp. 775–777). But it also highlights something distinct about play: While we routinely take the legitimacy of activities like working or studying for granted, adult play today remains contested and precarious, subservient to productivity norms. Take the following news story on a recent study by three economists. They observed that from 2004 to 2014, a significant and growing portion of noncollege-educated young men in the United States chose to displace wage labor time with playing video games. Given a lack of good job opportunities, this choice increased their life satisfaction. However, far from congratulating the young men on their economically rational preference maximization, the economists and reporting journalist paint this as a social problem: “if a historically vibrant portion of the population doesn’t feel as much desire to work, this could harm the economy’s future and the ability of government to use policy to create jobs”, or as one quoted economist put it: “That’s a big chunk of labor that could be used for something, and we’re not using it” (Swanson, 2016). Labor, historically viewed as divine punishment or the means to leisure, here is the taken-for-granted end of adult life, while adult leisurely play remains ever suspicious and in need of justification. The Protestant work ethic seems alive and well (Weber, 2010).

Maybe for this reason, most frames of legitimate adult play incorporate the full gamut of conditions, strategies, and alibis. German carnival for instance provides a well-established festival frame legitimized by tradition, huge play groups and lavish decoration establishing a clear framing, easy-to-follow scripts reducing required competence, the alibi of collective obligatory participation and intoxication, and ample awareness shields (masks, costumes, large crowds, loud music). It also demonstrates another fact: To not participate in an ongoing encounter of legitimate adult play is itself an embarrassing norm breach. Those whose persisting biographical identities clash with the identity of a carnival-goer but feel the social pressure to participate are easily recognizable by the most severe (and chagrined) acts of mock involvement, ironic meta-commentary, and role distancing. Or by leaving town for the duration.

Similarly, our analysis suggests multiple reasons why *games* are more suited for unembarrassed adult play than *free play*. Games are highly institutionalized and conventionalized and as such easily signaled and recognized; they come prelegitimized with imputed motives of recreation, family quality time, and so on; they are designed to be highly absorbing, leaving little mental reserve to become self-conscious (compared to the many pauses and stops of free play); they are highly scripted, ensuring smooth interaction and requiring little spontaneous creativity; and their rules offer established alibis for in-game actions.

Now nothing here is special. The conditions of embarrassment and means of deflecting it that obtain in adult play obtain for any social frame. If anything, the boundedness of play lends itself nicely to develop general theories. “Games seem to display in a simple way the structure of real-life situations” (Goffman, 1972, p. 32). The present essay is very much an exercise in this spirit.

As a sociological account, it necessarily foregoes individual difference and change. Psychology tells us that people have different degrees of embarrassability and that concern for how others perceive us can be unlearned (Miller, 1997). Furthermore, as a synchronic snapshot of one society, the present analysis is blind to cultural and historical differences and trajectories. How do “our” norms of legitimate adult play compare to those of other cultures and centuries? To what extent are the “ludification of culture” (Deterding, 2015a), the appearance of “emergent adulthood” (Arnett, 2007), “cultural neoteny” (Harrison, 2014), or postmodern “infantilization” (Bernadini, 2014) shifting the grounds of play and adulthood? To empirically trace the boundaries of embarrassment in adult play is to put such high-level descriptions of social change to the test. Just as Plato suggested that play reveals the natural dispositions of an individual, studying “what categories of persons become embarrassed in what recurrent situations” (Goffman, 1967, p. 109) may tell us much about the state of our societies.

## Notes

1. http://playpublik.de/en. Retrieved May 10, 2017.

2. http://ssrunners.org/. Retrieved May 10, 2017.

3. http://www.oregonlottery.org/ResponsibleGambling.aspx. Retrieved May 10, 2017.

## References

[bibr1-1555412017721086] Arnett J. J. (1998). Learning to stand alone: The contemporary American transition to adulthood in cultural and historical context. Human Development, 41, 295–315.

[bibr2-1555412017721086] Arnett J. J. (2001). Conceptions of the transition to adulthood: Perspectives from adolescence through midlife. Journal of Adult Development, 8, 133–143.

[bibr3-1555412017721086] Arnett J. J. (2007). Emerging adulthood: What is it, and what is it good for? Child Development Perspectives, 1, 68–73. Retrieved from https://doi.org/10.1111/j.1750-8606.2007.00016.x

[bibr4-1555412017721086] Ben-Yehuda N. (1990). The politics and morality of deviance: Moral panics, drug abuse, deviant science, and reversed stigmatization. Albany, NY: SUNY Press.

[bibr5-1555412017721086] Bernardini J. (2014). The infantilization of the postmodern adult and the figure of kidult. Postmodern Openings, 5, 39–55.

[bibr6-1555412017721086] Best J. (2003). Deviance: Career of a concept. Belmont, CA: Wadsworth.

[bibr7-1555412017721086] Bowman S. L. (2010). The functions of role-playing games. Jefferson, NC: McFarland.

[bibr8-1555412017721086] Boyd D. (2008). Taken out of context: American teen sociality in networked publics. Unpublished doctoral dissertation, University of California, Berkeley, CA.

[bibr9-1555412017721086] Boyle E. A. Connolly T. M. Hainey T. Boyle J. M (2012). Engagement in digital entertainment games: A systematic review. Computers in Human Behavior, 28, 771–780. doi:http://doi.org/10.1016/j.chb.2011.11.020

[bibr10-1555412017721086] Briggs C. L. (2007). Anthropology, interviewing, and communicability in contemporary society. Current Anthropology, 48, 551–580.

[bibr11-1555412017721086] Brown S. (2009). Play: How it shapes the brain, opens the imagination, and invigorates the soul. New York, NY: Avery.

[bibr12-1555412017721086] Busse K. (2013). Geek hierarchies, boundary policing, and the gendering of the good fan. Participations, 10, 73–91.

[bibr13-1555412017721086] Caillois R. (2001). Man, play, and games. Urbana: University of Illinois Press.

[bibr14-1555412017721086] Chapman A. Linderoth J. (2015). Exploring the limits of play: A case study of representations of Nazism in games In Mortensen T. E. Linderoth J. Brown A. M. L. (Eds.), The dark side of game play: Controversial issues in playful environments (pp. 137–153). London, England: Routledge.

[bibr15-1555412017721086] Cohen S. Taylor L. (1976). Escape attempts: The theory and practice of resistance in everyday life. Harmondsworth, England: Penguin.

[bibr16-1555412017721086] Copes H. Williams J. P (2007). Techniques of affirmation: Deviant behavior, moral commitment, and subcultural identity. Deviant Behavior, 28, 247–272. doi:http://doi.org/10.1080/01639620701233167

[bibr17-1555412017721086] Cresci E. (2016, 10 5). Norway’s PM caught playing Pokémon Go in parliament. The Guardian. Retrieved from https://www.theguardian.com/technology/2016/oct/05/norway-prime-minister-caught-playing-pokemon-go-parliament-erna-solberg

[bibr18-1555412017721086] Deterding S (2014). Modes of play: A frame analytic account of video game play. Unpublished doctoral dissertation, Hamburg University Retrieved from http://ediss.sub.uni-hamburg.de/volltexte/2014/6863/

[bibr19-1555412017721086] Deterding S. (2015a). The ambiguity of games: Histories, and discourses of a gameful world In Walz S. P. Deterding S. (Eds.), The gameful world: Approaches, issues, applications (pp. 23–64). Cambridge, MA: MIT Press.

[bibr20-1555412017721086] Deterding S. (2015b). The joys of absence: emotion, emotion display, and interaction tension in video game play In Proceedings of the 10th International Conference on the Foundations of Digital Games 2015 (FDG’15). Pacific Grove, CA: SASDG.

[bibr21-1555412017721086] Deterding S. Lucero A. Holopainen J. Min C. Cheok A. Waern A. Walz S (2015). Embarrassing Interactions In Proceedings of the 33rd Annual ACM Conference Extended Abstracts on Human Factors in Computing Systems (CHI’15 EA) (pp. 2365–2368). New York, NY: ACM Press doi:http://doi.org/10.1145/2702613.2702647

[bibr22-1555412017721086] Dunnigan J. F (2000). Wargames handbook, 3rd edition: How to play and design commercial and professional wargames. Bloomington, IN: iUniverse.

[bibr23-1555412017721086] Farrarons E. (2015). The mindfulness colouring book: Anti-stress art therapy for busy people. London, England: Boxtree.

[bibr24-1555412017721086] Goffman E. (1953). Communication conduct in an Island community. Chicago, IL: University of Chicago.

[bibr25-1555412017721086] Goffman E. (1967). Interaction ritual: Essays on face-to-face behavior. New York, NY: Pantheon.

[bibr26-1555412017721086] Goffman E. (1972). Encounters: Two studies in the sociology of interaction. Harmondsworth, England: Penguin.

[bibr27-1555412017721086] Goffman E. (1983). The interaction order. American Sociological Review, 48, 1–17.

[bibr28-1555412017721086] Goffman E. (1983a). Felicity’s condition. American Journal of Sociology, 89, 1–53.

[bibr29-1555412017721086] Goffman E. (1986). Frame analysis: An essay on the organization of experience. Boston, MA: Northeastern University Press.

[bibr30-1555412017721086] Granovetter M. S. (1978). Threshold models of collective behaviour. The American Journal of Sociology, 83, 1420–1443.

[bibr31-1555412017721086] Harrison R. P. (2014). Juvenescence: A cultural history of our age. London, England: University of Chicago Press.

[bibr32-1555412017721086] Harviainen J. T. Frank K (2016). Group sex as play: Rules and transgression in shared non-monogamy. Games and Culture, 1–20. doi:http://doi.org/10.1177/1555412016659835

[bibr33-1555412017721086] Heljakka K. (2013). Principles of adult play(fulness) in contemporary toy cultures: From Wow to Flow to Glow. Helsinki, Finland: Aalto University.

[bibr34-1555412017721086] Henricks T. S. (2015). Play and the human condition. Champaign, IL: University of Illinois Press.

[bibr35-1555412017721086] Jenkins R. (2008). Social identity (3rd ed). New York, NY: Routledge.

[bibr36-1555412017721086] Keltner D. Capps L. Kring A. M. Young R. C. Heerey E. A (2001). Just teasing: A conceptual analysis and empirical review. Psychological Bulletin, 127, 229–248. doi:http://doi.org/10.1037//0033-2909.127.2.229 11316012 10.1037/0033-2909.127.2.229

[bibr37-1555412017721086] King K. M (1990). Neutralizing marginally deviant behavior: Bingo players and superstition. Journal of Gambling Studies, 6, 43–61. doi:http://doi.org/10.1007/BF01015748 24242793 10.1007/BF01015748

[bibr38-1555412017721086] Lavenir G (2015, 4). Harmless gamers? Older gamers as a Trojan horse for the normalization of (video)games for adults. Paper presented at Tampere Game Studies Seminar Adult Play, Tampere, Finland.

[bibr39-1555412017721086] Lee J. (2009). Escaping embarrassment: Face-work in the Rap Cipher. Social Psychology Quarterly, 72, 306–324.

[bibr40-1555412017721086] Linderoth J. Mortensen T. E. (2015). Dark play: The aesthetics of controversial playfulness In Mortensen T. E. Linderoth J. Brown A. M. L. (Eds.), The dark side of game play: Controversial issues in playful environments (pp. 3–12). London, England: Routledge.

[bibr41-1555412017721086] McGraw A. P. Warren C. (2010). Benign violations: Making immoral behavior funny. Psychological Science, 21, 1141–1149. 20587696 10.1177/0956797610376073

[bibr42-1555412017721086] Miller R. S. (1997). Embarrassment: Poise and peril in everyday life. New York, NY: Guilford Press.

[bibr43-1555412017721086] Mills C. W. (1940). Situated actions and vocabularies of motive. American Sociological Review, 5, 904–913.

[bibr44-1555412017721086] Montola M. (2010). The positive negative experience in extreme role-playing In Proceedings of Nordic DiGRA 2010. DiGRA Retrieved from http://www.digra.org/wp-content/uploads/digital-library/10343.56524.pdf

[bibr45-1555412017721086] Montola M. Stenros J. Waern A. (2009). Pervasive games: Theory and design. Experiences on the boundary between life and play. Amsterdam, the Netherlands: Morgan Kaufmann.

[bibr46-1555412017721086] Moran G. (1979). Education toward adulthood. New York, NY: Paulist Press.

[bibr47-1555412017721086] OED Online. (2015). alibi, n., adv., and adj. Oxford, England: Oxford University Press Retrieved from http://www.oed.com/view/Entry/4978

[bibr48-1555412017721086] Oliver P. E. (1993). Formal models of collective action. Annual Review of Sociology, 19, 271–300.

[bibr49-1555412017721086] Olson E. A. Wells G. L. (2004). What makes a good alibi? A proposed taxonomy. Law and Human Behavior, 28, 157–176. 15141776 10.1023/b:lahu.0000022320.47112.d3

[bibr50-1555412017721086] Orbuch T. L. (1997). People’s accounts count: The sociology of accounts. Annual Review of Sociology, 23, 455–478.

[bibr51-1555412017721086] Peterson L. (2011). Values in play: Interactional life with the Sims. Gothenburg, Sweden: Chalmers University of Technology & University of Gothenburg.

[bibr52-1555412017721086] Poremba C. (2007). Critical potential on the brink of the magic circle In Situated Play, Proceedings of DiGRA 2007 Conference (pp. 772–778). Retrieved from http://www.digra.org/dl/db/07311.42117.pdf

[bibr53-1555412017721086] Ravenscroft N. Gilchrist P. (2009). Spaces of transgression: Governance, discipline and reworking the carnivalesque. Leisure Studies, 28, 35–49.

[bibr54-1555412017721086] Redmon D. (2003). Playful deviance as an Urban leisure activity: Secret selves, self-validation, and entertaining performances. Deviant Behavior, 24, 27–51.

[bibr55-1555412017721086] Rogerson M. J. Gibbs M (2016). Finding time for tabletop: Board game play and parenting. Games and Culture, 1–21. doi:http://doi.org/10.1177/1555412016656324

[bibr56-1555412017721086] Scheff T. J. (2000). Shame and the social bond: A sociological theory. Sociological Theory, 18, 84–99.

[bibr57-1555412017721086] Scott S. Hinton-Smith T Härmä V Broome K (2013). Goffman in the gallery: Interactive art and visitor shyness. Symbolic Interaction, 36, 417–438.

[bibr58-1555412017721086] Shut Up & Sit Down (2015). Review: Cards against humanity. Retrieved from https://www.shutupandsitdown.com/review-cards-against-humanity/

[bibr59-1555412017721086] Smith R. W. Preston F. W (1984). Vocabularies of motives for gambling behavior. Sociological Perspectives, 27, 325–348. doi:http://doi.org/10.3982/ECTA8577

[bibr60-1555412017721086] Stenros J. (2015a). Behind games: Playful mindset as basis for ludic transformative practice In Walz S. P. Deterding S. (Eds.), The gameful world: Approaches, issues, applications (pp. 201–222). Cambridge, MA: MIT Press.

[bibr61-1555412017721086] Stenros J. (2015b). Playfulness, play, and games: A constructionist ludology approach. Tampere, Finland: Tampere University Press.

[bibr62-1555412017721086] Sutton-Smith B. (1997). The ambiguity of play. Cambridge, MA: Harvard University Press.

[bibr63-1555412017721086] Swanson A. (2016, 9 23). Why amazing video games could be causing a big problem for America. The Washington Post. Retrieved from https://www.washingtonpost.com/news/wonk/wp/2016/09/23/why-amazing-video-games-could-be-causing-a-big-problem-for-america

[bibr64-1555412017721086] Thornham H (2009). Claiming a stake in the videogame: What grown-ups say to rationalize and normalize gaming. Convergence, 15, 141–159. doi:http://doi.org/10.1177/1354856508101580

[bibr65-1555412017721086] Turner J. H. Stets J. E (2006). Sociological theories of human emotions. Annual Review of Sociology, 32, 25–52. doi:http://doi.org/10.1146/annurev.soc.32.061604.123130

[bibr66-1555412017721086] Vines M. Linders A. (2016). The dirty work of poker: Impression management and identity. Deviant Behavior, 37, 1064–1076.

[bibr67-1555412017721086] Weber M. (2010). Die protestantische Ethik und der Geist des Kapitalismus: Vollständige Ausgabe. München, Germany: C.H. Beck.

[bibr68-1555412017721086] Williams R. (1988). Understanding Goffman’s methods In Drew P. Wootton A. (Eds.), Erving Goffman: Exploring the interaction order (pp. 64–88). Oxford, England: Polity Press.

[bibr69-1555412017721086] Wouters N. Downs J. Harrop M. Cox T. Oliveira E. Webber S…Moere A. V (2016). Uncovering the honeypot effect: How audiences engage with public interactive systems In DIS ‘16 Proceedings of the 2016 Conference on Designing Interactive Systems (pp. 5–16). New York, NY: ACM Press doi:http://doi.org/10.1145/2901790.2901796

